# Memoir on the Tumors of the Gums, Commonly Known as Epulis

**Published:** 1858-04

**Authors:** 


### ARTICLE V.

*Memoir on the Tumors of the Gums, commonly known as Epulis.* By Doctor Saurel. (Translated from the French for the American Journal of Dental Science.)

Caries and necrosis of the alveoli, or of the maxillary bones, figure among the causes to which the production of epulis is attributed. Without denying the reality of these causes, it seems to me that, if they exist, they are at least rarer than is generally supposed. In examining the different cases of epulis attributed to caries of the maxillæ, I have ascertained that, most frequently, the tumor had preceded the alteration of the bones, and that it presented characters widely different from fungous tissue. This remark is especially applicable to two cases reported by M. Rigal (de Gaillac) in the *Revue Medicale,* for the year 1827, and in which the tumors presented a truly carcinomatous character. The following, though very incomplete, belongs perhaps to the variety of epulis under consideration.

CASE IV.—*Fungous Epulis, with caries of the lower jaw, cured by cauterization.* (By JOURDAIN.)

"In 1767," says the author, "a girl of about eighteen years of age called on me. Among many eroded teeth which she had in the lower jaw, a large molar of the left side was extremely carious, the greater part of its crown having been destroyed. This tooth had occasioned several fluxions, terminated by parulis, which opened sometimes spontaneously, and sometimes by the aid of cataplasms and emolient gargles; but, as the tooth had not been removed, the parulides remained fistulous, the edges turned out, became fungous, and there resulted a fleshy mass wider and thicker than a piece of three livres, more annoying than painful, shaped like a cauliflower, and bedewed with a tenacious humor. This excrescence appeared to involve the cheek and the external plate of the maxilla. It extended so far beyond the teeth, that the patient bit it while eating, causing several times a sort of hemorrhage. On passing a curved sound around this excrescence, I ascertained that it was directly adherent by its middle to the gum itself, without having such a neck as to admit of the use of a ligature.

"I thought proper to commence the treatment by the extraction of the roots of the carious teeth. The extremities of each were enveloped in a hypersarcosis as large as a pea, terminated by a pedicle, which I supposed was implanted directly upon the substance of the bone. The intermediate septum of the roots was completely destroyed, the maxillary bone was sound on the lingual surface, but on the buccal surface riddled with holes like a sieve. The sound traversed it, and entered the epulis.'' The author treated this tumor by the actual cautery. He applied first a red-hot button to its centre. A few days after this first operation, he withdrew a portion of the external alveolar layer which was carious. He applied the fire a second time with a cutting cautery, which provoked new exfoliations of the bone, and completed in a short time the cure of the epulis.

The following is a case of epulis with necrosis of the maxilla, recorded, like the preceding, in the article in the *Dictionnaire des Sciences Medicales.* The reader will judge how far the development of the tumor could be attributed to a pre-existing necrosis of the maxilla. As far as I am concerned, I should be rather disposed to believe that the necrosis succeeded the epulis, instead of causing it.

Case V.—*Epulis, with necrosis of the lower jaw.* (By Manget.)

''Jean-Nicolas Ma-Chaick, surgeon, was consulted, in 1690, upon an excrescence of flesh, of considerable size, on the lower jaw of the right side, which had originated among the canine teeth of the sexagenarian lady. This excrescence had so increased, during the space of five years, that it was larger than a hen's egg, so that these two teeth, between which it originated, like a superficial caruncle of flesh, separated from one another a finger's breadth, and started from their alveoli, so that the mouth was deformed by perpetual gaping, and could not be closed at all.'' This epulis was treated by a ligature of very flexible iron-wire. On the ninth day, little laminæ, which separated spontaneously from the maxillary bone, did not permit the ligature to be steadily tightened, as had been done previously every day. It was determined then to cut away what remained of the pedicle of the tumor, and on the part occupied by the epulis a desiccative powder was applied. Three days after could be seen the base of the wound, whence issued, without any pain, nine spiculæ of bone, and the patient was soon completely cured.

Fungous tumors analogous to the preceding, are sometimes formed without previous dental caries, or alteration of the alveoli. Almost always, it is true, the teeth in the neighborhood of the epulis, are straggling and loose, but they are perfectly sound; which shows that the disease has commenced in the gums, or in the alveolo-dental periosteum.

The case which I am about to report, is remarkable in several respects, but especially for the considerable development of fungous tissue, which had invaded the two alveolar arches.

CASE VI.—*Fungous excrescences of the gums and of the alveolo-dental periosteum.—Extirpation.—Cure.* (By LEONARD KŒCKER.)

"A man, sixty years of age, had in his mouth, on a level with each of the dental arches, a fungous tumor, which had been developed for thirty years. This tumor occupied exactly the place of the gums, and extended before and behind the teeth of each jaw, so that their free border alone was visible. This disease had begun thirty years before, and had resisted all the means employed for its relief; it kept up a continual irritation of the mouth, and caused acute pain.

"M. Kœcker thought that the tumor was formed at the expense of the gums and alveolo-dental periosteum, and that the sole means to be employed was, the simultaneous removal of the teeth and the tumor. He began by taking away the patient's remaining twenty-nine teeth; this operation gave little pain, for most of the dental nerves were destroyed; then the next day he removed with strong scissors the whole diseased tissue. The cure has been complete. Two years after the operation, the mouth is in good condition; mastication and pronunciation are performed with sufficient ease.''

The result of the operation, which has been followed by a complete cure, although cauterization was not employed, proves that here we had to deal only with excrescences of the gums, and perhaps of the alveolo-dental periosteum, in which the osseous tissue had nothing whatever to do. The long duration of the disease, in spite of the acute pain it caused, proves its benign nature. In the following case, the tumor was undoubtedly of the same nature, although it was formed quite rapidly.

Case VII.—*Fungous Epulis of the upper jaw, operated upon by excision, followed by cauterization.* (By M. Guersant.)

''M. Guersant brought to the amphitheatre a little girl, who presented quite a curious example of epulis. She had, upon the upper jaw, a soft bloody tumor, having the aspect of fungous tissue, and situate on the external face of the maxilla. This vegetation sprouted up between the molar teeth, after having originated apparently in the thickness of the alveolo-dental periosteum. Was it of a cancerous character? No! but nevertheless, M. Guersant thought that it was necessary to remove it, to prevent extension. He decided not to cut the cheek, and performed the operation at two separate times. He removed one day three loose teeth; then, eight days after, while the child was under the influence of chloroform, he cut away the epulis with scissors and a bistoury, then boldly cauterized the base of the tumor with an iron heated to whiteness. This tumor was three months old. Eight days after the operation, the child was very well.''

The author above quoted, says nothing of the structure of the removed tumor; still its external characters permit us to consider it fungous. I do not know how far the excision of the bone was necessary, for the adhesions of the epulis were not deep. An excision of the tumor alone, followed by cauterization, would have been, it appears to me, perfectly sufficient.

The six observations, which I have just copied, all belong to one and the same species of tumor, which merits the name of *fungous epulis*, for it is essentially constituted by the more or less rapid development of reddish fungosities, bleeding readily, tearing of their own accord, and furnishing a sero-purulent exudation, more or less fetid. Their exclusive seat is the tissue proper of the gums, and perhaps, also the alveolo-dental periosteum. If there is an osseous lesion, it exists only as a cause or as a complication, but the tissue of the new formation never invades the bone itself. These tumors are generally indolent, or, if they cause pain, they have not the lancinating character we shall have to point out in another species of epulis. The inconveniences which their presence occasions are common to other forms of the disease; we shall presently speak of them. Although the anatomical structure of these tumors has not been sufficiently studied, and though nothing is said of it in the different reports of cases which we have quoted, we can nevertheless, positively affirm, that they are benign. They do not regerminate when they are completely removed, after their cause has been destroyed.

Common as fungous tumors of the gums appear to be, those which are said to be formed of erectile tissue, and the first description of which is due to Marjolin and A. Berard, are rare. Among the cases which I have found recorded in authors or journals, but one relates to this species of epulis, to which I shall give, to distinguish it from others, the name of *vascular* or *erectile epulis.* Nowhere have I found positive indications concerning their structure; all the authors who mention them are content to say, that their organization *appears* to be the same as that of erectile tumors. It is desirable that surgeons, who may have occasion to observe these tumors, should not neglect to attend to their anatomical examination.

The following is my solitary case.

CASE VIII.—*Erectile Epulis developed on the internal face of the superior alveolar arch.—Ligature and cauterization.* (By BLANDIN.)

M. le Professeur Blandin removed, on the 24th of December, 1847, a little fungous tumor* of the palatine vault, from a woman forty-one years of age. This tumor, which originated two years before, had occurred without any known cause, and had gradually acquired the size of a blackbird's egg. Situated on the anterior and external part of the vault of the palate, it presented itself under the form of a mushroom. At first sight it appeared to be sassile; but, on circumscribing it with the finger, it was ascertained that it was borne up on very delicate pedicle, implanted near an alveolus. Red, compressible, indolent, but bleeding under the influence of an irritating contact, this fungus gave to the touch the sensation of trembling and throbbing peculiar to fungous tumors, and particularly to the fungous tumors of the palatine vault.

"In the case now under consideration, the cause of the disease was absolutely unknown. It was only known that the tumor was a simple pedunculated epulis perfectly independent of the bones. Now, under such circumstances we need not hesitate. We must anticipate a possible degeneration by operating." The tumor being pedunculated, M.

* It is time to give to the terms *fungus, fungous tumor, fungosity,* their veritable signification. The vascular and erectile tumors, such as that we are now considering, are very distinct from true fungous tumors. Fungosities are fleshy, soft, spongy, vegetatious, shaped like a mushroom, which are often developed on the surface of wounds or of ulcers. According to Ch. Robin, they are composed—1st, of amorphous granular matter, of the very abundant, especially when they are soft; 2d, of fibro-plastic elements; 3d, of fibres of cellular tissue, slender, pale, interwoven, rarely in bundles, and glued together with amorphous matter; 4th, of capillaries, less abundant as the color and softness of the product seem to indicate, at other times very numerous in the bleeding fungosities; 5th, sometimes we find these *granular inflammatory globules,* or even globules of pus only on the surface of the tissue. If the tumor issues from a cancer or an epithelium ulcerated, we find their elements in it. The tumors formed by these fungosities alone deserve the name of *fungous tumors.* The first of the kinds of epulis admitted by M. Robin belongs only to this order of morbid productions.

Blandin had recourse to the ligature ; but the pedicle was so slender that it was scarcely compressed by the knot of the thread, when it was severed, and the tumor dropped off.

It is to be regretted that the author has not given details of the structure of this tumor ; he is content to say that "the tumor, examined with care, presents none of the elements peculiar to tumors of a malignant character." The termination of the vase is equally imperfect, and we are ignorant of the completeness of the cure.

Vidal (de Casis) has studied vascular tumors of the gums in connection with degenerations of the lower jaw ; but he has confounded in a common description the vascular tumors of the gums, properly so called, and those of the inferior maxilla ; so that it is very difficult to distinguish, in his somewhat overdrawn picture, what belongs to either of the classes of tumors. These are the terms in which he sums up their characters :

"These degenerations are observed under the form of erectile tumors, we remark them especially in the portion of the jaw which corresponds to the alveolar arch. In some cases, the affection is first displayed in the tissue of the gums, and has only consecutively invaded the neighboring parts.

"The patient complains, in the first instance, of a dull continued pain, with temporary exacerbations, during which the pain becomes acute, lancinating, and darts in the direction of the inferior dental nerve, either towards its origin or towards its terminal branches, which results probably from the compression of some filaments of the dental nerve, or of the trunk itself.

"Soon the surgeon can determine the mobility or the loss of the teeth. Erectile tissue takes the place of the osseous tissue, the gums and the mucous membrane which envelops the jaw are changed and undergo vascular degeneration. The tumor of a livid red color swells, and becomes darker during the cries and struggles of the patient; it then diminishes, and may, under the influence of pressure, undergo a still more considerable subsidence.

"In proportion as the tumor acquires a greater development, the functions of the jaw and of the tongue are more embarrassed; as greater number of teeth become loose, some fall. Gradually, the erectile tissue ends by invading the whole thickness of the body of the jaw.

"The morbid mass, which presents more or less decided protuberances, ends by ulcerating on one or more points, hemorrhages frequent, and more and more abundant, a habitual sanious dribbling, produce a profound alteration of the constitution, and then death."

The three species or varieties of epulis which we have already studied are not the only kinds; there exists several others. That with which we shall now occupy ourselves differs from the preceding by numerous characters, among which we must specify its hardness, its rounded form, its pale or red color, its compact structure, &c. A very incomplete report of a case recorded in the transactions of the Chirurgical Society, will be followed by two others which belong to me, and in which I believe nothing essential is wanting. The comparison of these reports will enable us to establish the character of fibrous epulis.

CASE IX.—*Epulis of the lower jaw, removed by excision.* (By M. GUERSANT.)

"M. Guersant presented to the Surgical Society, (at the session of May 11th, 1853,) a child ten years old, endowed with a good constitution, who had, on the upper border of the lower jaw on the left side, a consistent, movable, bloody tumor, of quite a lively red color. M. Guersant having some hesitation in diagnosticating the nature of this affection, inquires what treatment it is best to pursue. MM. Gosselin, Forget and Lenoir, who have observed similar tumors, advise the removal with the tumor of the bone on which it rests.

At the session of June 22d, M. Guersant again presents this young boy. The operation has been performed by the aid of curved scissors. The whole tumor and the part of the alveolar border to which it adhered, were removed at a single clip. On the bottom of the notch resulting from the operation, appeared a tooth of the second dentition. It was necessary to divide the labial commissure obliquely downward, to be able to manœuvre the instrument. Cicatrization took place by first and second intention.

It is to be regretted that M. Guersant has not informed us of the structure of the removed tumor; we should know then if it really sprung from the maxillary bone, and if it was indispensable to cut that away. We shall return to the question under the head of treatment.

Case X.—*Fibrous Epulis of the lower jaw, successfully treated by extirpation, followed by cauterization.* (By the Author.)

J. B. Roux, farmer, aged sixty-two years, living at Montpellier, faubourg Boutonnet, No. 108, was directed to me about the close of the month of December, 1853, by my fellow-practitioner, Doctor Fave. This man, of a lymphatico-bilious temperament, and of a constitution worn out by wretchedness and labor, has been frequently attacked by thoracic diseases. At this time he suffered from chronic catarrh, accompanied by pains in the legs and marked exhaustion. His complexion is pale and sallow, and his muscles are flabby. The disease for which he comes to consult me is a tumor which he has in his mouth, and which has been there for several months.

This tumor, of the size of a walnut, of a form a little oblong transversely, occupies the left lateral portion of the lower jaw, above which it rises. It adheres to the gum in the part corresponding to the second incisor and the canine of the left side, which it covers, and above the level of which it rises. Its upper part presents a transverse groove, impressed by the upper teeth, which rest upon it when the jaws are closed; below, it reaches the labio-gengival cul-de-sac. This tumor appears very hard to the touch; its color differs little from that of the gums, that is to say, it is of a pale rose tint: the furrow made by the pressure of the upper teeth is of a grayish white. It adheres strongly to the tissue of the gums, but it possessed a degree of mobility, and by lowering it and moving it alternately from right to left, it is easy to perceive that it is attached by a pedicle of less diameter than its base. Its surface appears mammilated, and around it are some vegetations of the same nature and color, insinuating themselves between the adjoining teeth, and protruding upon the posterior aspect of the jaw. The second incisor has been pushed a little backward by the tumor, and is somewhat movable.

The tumor is but slightly painful; it is only at remote intervals that the patient feels some trifling pain; it can be pressed in all directions without developing tenderness. The submaxillary glands are perfectly healthy, showing no indication of engorgement. The same is true of those in the cervical region. The patient demands to be relieved of this tumor, which annoys him greatly, and hinders him from eating with satisfaction; indeed, the complete closure of the jaws having become impossible, the man is reduced to eating nothing but soup, or compelled to masticate imperfectly and with difficulty.

This morbid production originated without known cause; there is no carious or diseased tooth in its neighborhood, and he does not remember ever to have had his gum wounded. The man does not smoke, he lives regularly, though wretchedly, has never had any other tumor, and has not heard of any member of his family having had any thing of the kind. He was not aware of the existence of the tumor until it had attained the size of a pea; it remained stationary for more than six months; after that it progressively increased without presenting any special phenomena.

The general condition of the patient not being very satisfactory, I thought it better to defer the operation until that was improved, and his strength had returned. Meanwhile I put him upon snail soup, bitters, decoction of Iceland moss, &c. On the 3d of February, 1854, the patient finding himself much better, and ardently desiring the operation, I decided to perform it in the presence and with the aid of my friend, Doctor Girou.

The patient being seated in front of a window, the tumor was dissected and removed with the bistoury. I took care to go beyond its base and act upon the sound portions of the gum. A little blood ran from the lower portion of the wound; this hemorrhage yielded to light compression by the finger. On the removal of the principal tumor, in order to destroy completely the numerous vegetations about its base, I found it necessary to remove the second incisor and its neighboring canine. With the scissors and the bistoury I could then excise all the suspected parts. An olive-shaped cautery, heated to whiteness, was then passed backwards and forwards over the wound, so as to destroy not only the remaining portions of the morbid product, but also the periosteum.

This little operation was well borne by the patient, who lost at most 60 grammes (15 ounces) of blood. Lotions and applications of cold water were immediately made. (*Anti-spasmodic potion.*) No accident followed the operation. A slight inflammation, followed by sloughing of the eschars formed by the cautery, took place in the next few days. Fleshy excrescences soon followed by a cicatrix, were developed upon the spot where the tumor had been. The cure was delayed only by the presence of a little superficial necrosis of the maxilla. It was perfect about the first of March. A solid cicatrix, of a mucous appearance, covered the edge of the maxilla, and the general condition of the patient was much improved. Since then I have repeatedly seen the patient and he has had no relapse.

The removed tumor was very firm; the bistoury divided it with difficulty; its section of a pearly white, reddened in points, was brilliant; pressure caused no exudation of liquid more than the act of scraping with the bistoury. All its parts were exactly alike.

CASE XI.—*Fibrous Epulis of the lower jaw, cured by extirpation, followed by cauterization.* (By the AUTHOR.)

The widow Hubert, aged sixty-eight years, living in Montpellier, Rue de la Garenne, No. 5, was brought to me on the 28th of October, 1856, by the wife of the last named patient. About five years ago a tumor appeared upon her lower jaw, beginning as a little protuberance upon the gums, between the incisors of the right side, following what is called a defluxion on the gums, but without the presence of any carious tooth.

The tumor, of the size of a common walnut, a little flattened from before backward, presenting a rounded circumference and a slightly mammillated surface, is of a lively red color. It bleeds easily, but is completely indolent. It adheres by its lower border and its posterior surface to the lower alveolar border, making by its presence the position of the right incisors and canine. When the jaws are approximated, this tumor, insinuating itself between the dental arches and the lips, throws the latter far forward. It annoys the patient by its presence and its partial obstruction to mastication, but it has never caused pain. The general condition is good enough, though this woman is worn with age and toil.

On the 30th of October, I operated in presence of Dr. Girou. A curved bistoury, inserted between the tumor and the alveolar border, easily detached it. Some excrescences of the gum, which were hidden by the principal tumor, were removed by the scissors. The second incisor tooth, the neck of which was surrounded by these excrescences, was, although sound, extracted with the forceps. A little blood oozed from the wound, chiefly from the alveolus of the removed tooth; it yielded to cauterization with a hot iron upon the site of the tumor. The pain experienced by the patient was trifling.

The next day there was a very slight swelling of the gums and lips, but neither hemorrhage nor pain. Four days afterwards, the eschar was detached. At the end of fifteen days cicatrization was complete, except a little point in front of the gum, on the site of the tumor.

On the 6th of December, the patient returned. There was a little fistulous opening on the gum, in front of the right lower canine, the crown of which is worn, but healthy. A probe introduced into the fistula reached this tooth, which was immediately removed. Twenty days later the cure was complete. A pale, solid cicatrix existed upon the site of the tumor, and of the extracted teeth. The cure was not deceptive.

The tumor, examined shortly after the operation, had lost its red color and assumed a yellowish white tint. It was hard, and could neither be compressed nor crushed between the fingers. It creaked under the scalpel, was white, lardacious and somewhat iridescent. On moving the cutting edge of the instrument across the section, nothing like cancerous juice exuded. The most attentive examination discovered neither vessels nor bony matter. It should be added, that this tumor was evidently implanted upon the fibrous tissue of the gums, which it partly resembled in appearance.

The most perfect resemblance in seat, symptoms, consistence, structure, &c., exists between the tumors of my two patients; it is therefore impossible to confound fibrous epulis with the other species. Its general characters may be summed up as follows:

Absence of appreciable, general or local causes. Very slow progress of the morbid product, which may last months and years without ulcerating. Tumor of a rounded form, a mammillated surface, a color rosy or bright red, sometimes bleeding, hard, incompressible, completely indolent and usually movable. Its pedicle is implanted on the gums, and sends prolongations between the teeth, which are displaced and loosened. The section of the tumor, somewhat nacreous, homogeneous, and presenting the appearance of fibro-plastic tissue; no juice can be expressed, and it cannot be crushed. The results of operations prove that these tumors have no communication with the osseous tissue; it is therefore unnecessary to cut away any portion of the maxilla.

Can tumors of this sort degenerate, as the good Ambrose Paré will have it they do, and as a goodly number of authors have subsequently admitted? This degeneration is not more possible for epulis than for other tumors. New tissues may be superadded to and substituted for those which already exist, but there is never a true degeneration. Still it is incontestible that the structure of recurring tumors is not always the same as that of the primitive swellings—this is strikingly evident, at the end of certain operations for cancer. The following case, which we borrow from M. Marfan, will tend to establish this point. Let us remark before hand that, in this case, we have no longer to deal with a perfectly benign tumor, since it returned. Its osseous structure is a circumstance the more remarkable, from the fact observed by the author, that it was very movable and easily detached; which proves that the new bony tissue was not continuous with that of the maxilla.

CASE XII.—*Osseous Epulis of the upper jaw, following dental caries, treated by ligature.—Recurrence.—Extirpation and cauterization.—Cure.* (By M. MARFAN.)

Marie Mazières, forty-five years of age, of a strong constitution and sanguine temperament, came to consult M. Marfan concerning a tumor which had its seat on the alveolar border of the upper jaw. The patient stated, that ten years before she was attacked with dental caries, which invaded successively the three first molars. The first and third were in turn destroyed by this disease, without giving rise to very acute pain. The second, on the contrary, occasioned very intense neuralgia. The caries, as with the first, removed the crown; the root remained. A year after, she observed, in the centre of the alveolus, a little rounded tumor of the size and shape of a lentil It increased almost imperceptibly for about eight years; but, after this period, its volume increased sufficiently to become troublesome. At this period, the tumor was pedunculated. The patient, by the advice of a physician, used a ligature of silk upon it; but, shortly after its fall, she observed the disease recur and increase more rapidly than ever.

It was a year after this little operation that the patient came to consult M. Marfan. The tumor was then five centimetres broad, four long and two thick. It was flattened and divided into three lobes; its color was that of the buccal mucous membrane; it was hard, resisting, and very movable. The maxillary sinus, when explored, presented no pathological condition. All these circumstances, together with the absence of lancinating pain, induced M. Marfan to believe that he had an epulis to deal with and to counsel an operation.

The tumor being dissected from its adhesions and its pedicle cut, the alveolus of the second molar was discovered enclosing a small, hard, movable body, which was the remainder of the root of the tooth, and which was taken with forceps. This circumstance decided M. Marfan to cauterize the alveolus with red hot iron. Seven days afterwards, the wound was completely cicatrized; but a little spicula, lodged under the cicatrix inducing pain, it was necessary to make an incision in order to extract it. From this time the patient had no further trouble.

The tumor was composed of a tolerably delicate pellicle, having all the characters of the buccal mucous membrane. Its interior was formed of a bony tissue, with large laminæ, well organized, and permitting the exudation of a rather thick, whitish liquid. The microscope revealed nothing but epithelial cells.

We now come to the species of epulis, which is at once the gravest and the most imperfectly studied. Confounded by some, especially Léveillé, with cancer of the jaw and tumors of the maxillary sinus; attributed by others to caries of the maxillary bones, this disease possesses characters of its own, and deserves to be studied by itself. Its resemblance to certain forms of cancer, the numerous vegetations it sends, and the facility with which it returns, induce me to call it *carcinomatous epulis.* The following are cases of this morbid species:

Case XIII.—*Carcinomatous Epulis of the lower jaw, treated by excision.—Recurrence.—Cauterization, followed by cure.* (By Brouillard.)

A young lady, eighteen years of age, of a delicate constitution, formerl yrachitic, had a fleshy excrescence, which, from the internal face of the left side of the lower jaw, when it took root beneath the first and second molar teeth, extended as far as the inner face of the right side. This tumor, occupying almost the entire space of the arch of the lower jaw, had displaced from it the tongue, and kept it applied to the palate, so that the patient spoke, ate and swallowed with difficulty. The upper surface of this fungosity, somewhat resembling a great flattened horse chesnut, was half opened by a deep and irregular crevice, whence issued a bloody oozing. Its pedicle was not larger than a twenty-four sous piece, and its mass was free and floating in the mouth. The patient suffered almost continual lancinating pains, which were often aggravated during the night, the interior of the bone seeming to be their principal seat. This tumor was three years old; its origin was referred to a laceration of the gums by a piece of nutshell.

It would have been easy to apply the ligature to the tumor, but the surgeon, after having extracted the two first molar teeth, which were very vacillating, thought proper to use the bistoury. The tumor was removed without difficulty, astringents and compression served to arrest the hemorrhage. On the next day, and for eight days after, the wound was cauterized with *lapis infernalis;* but the condition of the wound underwent no favorable change. Its repullation was so rapid that in the evening it could not be perceived that the caustic applied in the morning had at all diminished the elevation of the flesh. It was always hard, unequal, painful, and bleeding at the slightest touch. M. Brouillard then resorted to the actual cautery, (of silver.) The fall of the eschar took place on the eighth day. It left a hollow surface, without regerminating vegetations as before. Still, the aspect of the wound was not yet satisfactory; the bottom was hard and bleeding; slight lancinations were felt, and the fungous growths seemed ready to re-appear. A second application of the cautery was considered the more necessary as it was evident that the roots of the disease were planted in the bone, and that it could not be destroyed without inducing exfoliation. The eschar which succeeded this second application did not fall till the twelfth day, but the vicious local action was totally destroyed; the wound was covered with healthy granulations; the exfoliation of the bones took place almost insensibly, and the cure was perfect two months after the second application of fire.

Although we have no information relative to the structure of the removed tumor in the case just cited, the clinical details are sufficiently precise to leave no doubt of the carcinomatous nature of the disease. It is certain, as the author has remarked, that the roots of the disease were planted in the bone, and that a partial destruction of the latter was necessary to prevent a new growth. Besides, the lancinating pains felt by the patient, and the rapidity with which the fungosities was reproduced, render the diagnosis certain.

Is the same true of the following case, which we find recorded in the surgical works of Desault? I am induced to believe it, although the recital leaves much to be desired.

Case XIV.—*Fungus of the lower jaw removed by Desault.—Death.*

Françoise Méton, 34 years of age, of a strong and robust temperament, but habitually subject to fluxions and diseases of the teeth, was attacked, in 1790, by pains, which having had their seat at first in the head and the right arm, passed to the right side of the lower jaw, became fixed there, and greatly tormented the patient. At the same time, an indolent tumor, not painful to the touch, although its centre was subject to continual lancinating throbs, rose above the ramus on this side, and gradually extended inwards and outward. The teeth, almost all carious, began to loosen, and were successively extracted, as the pain which they excited rendered their continuance in the mouth insupportable. The gums swelled, the tumor increased, occupied half the bone, interfered with deglutition and the articulation of sounds, rendered the opening of the mouth painful, broke, gave issue to a little sanious pus, and became the seat of two fistulas, one above, and the other outwards. Upon their orifices sprang up fungosities; at the same time the bone became carious below and in the middle of the tumor, several portions of it became detached, and under the lining membrane of the mouth formed little deposits, which, being open, allowed them to pass; the breath became fetid, often insupportable.

Such, for two months, had been the condition of the patient, when she came to the Hotel Dieu to consult Desault. The examination of the parts revealed a fungus rising above the right side of the jaw, extending from the place which the last molar occupies to that which in the normal state the canine fills, enlarging from behind forwards, being nearly three inches in this direction, and complicated with necrosis of the subjacent portion of bone, which was perceived to be denuded by passing a probe through the fistulous openings.

Extirpation being considered necessary, Desault practiced it two months after the entrance of the patient. Two semilunar incisions encircled the tumor; then, to remove the bony portions, he employed a strong, thick instrument, curved in the form of a pruning-knife, which, plunged deeply into either incision, completely isolated the tumor. Considerable hemorrhage having supervened, pledgets of charpie were introduced into the wound and then removed to make room for the actual cautery, which was passed several times over the whole extent of the wound.

The next day, no pains, little swelling, every prospect of success; but the fifth day, pains of the kidneys, diarrhea. At the same time, swelling of the tonsils, difficult deglutition, fever. These symptoms go on steadily increasing, and the patient succumbed on the tenth day, from general prostration.

The circumstances which induce me to consider the tumor removed by Desault a carcinomatous tumor, are, first, the appearance, on the border of the jaw, of a tumor insensible to the touch, but occasioning continual lancinating pains, which constantly increased and determined a profound alteration of the bone on which it was seated; and in the second place the facility with which the tumor was *isolated completely*. These characters appear to me sufficient to prove that this was not a cancer or other tumor developed in the body of the jaws.

---

### ARTICLE VI.

*Salivary Calculus.* By A. C. CASTLE, M. D.

